# Supplementary material for: Transcriptome analysis and connectivity mapping of Cissampelos pareira L. provides molecular links of ESR1 modulation to viral inhibition
Source: Sci Rep. 2021 Oct 11;11:20095. doi: 10.1038/s41598-021-99444-0 (PMC8505412; doi:10.1038/s41598-021-99444-0)
Supplement: Supplementary file 1 — Supplementary Information 1. [file 41598_2021_99444_MOESM1_ESM.pdf]

Supplementary material

Transcriptome analysis and connectivity mapping of *Cissampelos pareira* L. provides molecular links of ESR1 modulation to viral inhibition

Madiha Haider[1,2¶] Dhwani Dholakia[1,2¶] Aleksha Panwar[5] Parth Garg[3] Atish Gheware[1,2] Dayanidhi Singh[1,2] Khushboo Singhal[1,2] Shaunak A Burse[1,2] Surekha Kumari[6,2] Anmol[6,2] Arjun Ray[3] Guruprasad R. Medigeshi[5] Upendra Sharma[6,2] Bhavana Prasher[*1,2,4] Mitali Mukerji [*1,2,4]

[1]Genomics & molecular medicine, CSIR-Institute of Genomics and Integrative Biology, Delhi, India; [2]Academy of Scientific and Innovative Research, Ghaziabad, Uttar Pradesh, India; [3]Indraprastha Institute of Information Technology, Delhi, India; [4]Centre of excellence for Applied developments of Ayurveda prakriti and genomics, CSIR Ayurgenomics Unit TRISUTRA, CSIR-IGIB; [5]Translational Health Science and Technology Institute, Faridabad, Haryana, India; [6]Chemical Technology Division, CSIR-Institute of Himalayan Bioresource Technology, Palampur, Himachal Pradesh 176 061, India

*Corresponding Authors

Email: bhavana.p@igib.res.in (BP), mitali@igib.res.in (MM)**

** Current address: Department of Bioscience & Bioengineering, Indian Institute of Technology Jodhpur, NH 62, Karwar, Rajasthan 342037

¶Equal contribution by these authors

Keywords: *Cissampelos pareira*, ESR1, Estrogen receptor, Dengue, Antiviral, connectivity map

Materials and methods:

Cell viability assay:

Toxicity of the extract at the aforementioned doses was done using MTT (HiMedia, Cat# TC191) assay. MCF7 cells were seeded in 96 well plates and the extracts were added. The treated cells were kept at 37˚C and 5% CO_2 for 24 hours. The media was then removed and MTT at a final concentration of 20µg per well was added. The cells were then kept in the incubator for 4 hours at 37˚C and 5% CO_2. The reduced formazan crystals were then dissolved in 100µl DMSO and kept at room temperature in the dark for 15 minutes. The absorbance was measure at 560nm using Tecan plate reader Infinite200 Pro. Each concentration was analyzed in triplicates and the experiment was repeated three times. For this assay paclitaxel at a concentration of 10µg was used as positive control.

Phosphorylation of EIF2α by Cipa:

To check the ratio of pEIF2α: EIF2α first in cell western was done using antibodies against pEIF2α (ProSci, Cat no. 79-226) and EIF2α (Abbkine, Cat no. ABP51245). Secondary antibodies from LI-COR, Anti-Rabbit 680 (Cat no.926-68023), Anti-mouse 680 (Cat no.926-68022) and anti-rabbit 800 (Cat no. 926-32211) were used. The MCF7 cells were seeded in a 96 well plate and treated with the extract for 24 hours. The cells were washed with PBS and fixed using 3.7% formaldehyde. After permeabilizing the cells with 0.1% triton X, blocking of the proteins was done using 3% BSA for 1.5 hours. The cells were incubated with primary antibodies overnight at 4 degrees and washed with 0.01% tween 20, followed by incubation with secondary antibodies for 2 hours. The plate was read in LI-COR Odyssey Infrared reader after thorough washing and drying. The infrared fluorescence was measured by manual selection of the wells. The values were noted and the ratio was calculated.

HPG global protein synthesis assay:

To assess the effect of the extract on global protein synthesis, Click-iT™ HPG Alexa Fluor™ 488 Protein Synthesis Assay (Cat no. C10428) was done according to the manufacturer's protocol. Briefly, Cells were allowed to grow overnight and the media was changed to DMEM high glucose without cysteine and methionine containing HPG. The cells were co incubated with 500µg and 1000µg extract for 3 hrs. As a positive control for protein synthesis inhibition, cycloheximide was used at the concentration of 10µg. The cells were washed with PBS, fixed with formaldehyde and permeabilized using triton-X. Substrate to HPG was added and the cells were incubated for 45 mins, counterstained with DAPI and the fluorescence was measured using High content imaging

Collection of plant material and preparation of extract:

Whole plant of C. pareira was collected from the Palampur, HP, India (alt. 1350 m). The identification of the plant material was done by a taxonomy expert in CSIR-IHBT, Palampur and a voucher specimen (no. PLP16688) was deposited in the herbarium of CSIR-IHBT, Palampur, HP-176,061, India.

Chemicals and reagents:

For UPLC analysis, formic acid was obtained from S. D. Fine Chemicals Ltd. (Mumbai, India) whereas methanol and water (LC grade) were purchased from J. T. Baker (Mallinckrodt Baker Inc., St. Louis, MO, USA).

The pure molecules from roots of the plant were isolated as reported by us recently (3).

Figures:

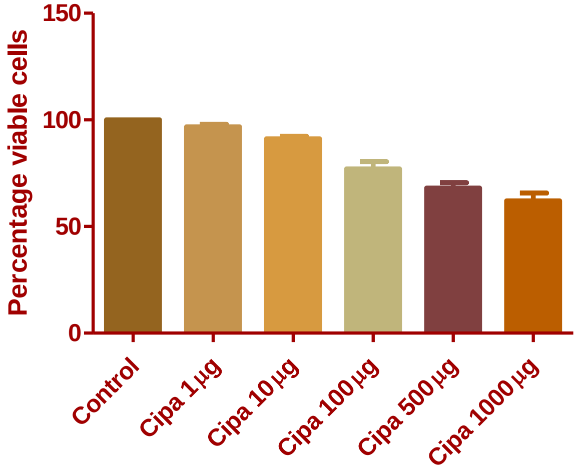

Fig. S1. Cipa doesn't show toxicity in MCF7 cells, except at higher concentrations. MTT cell viability assay shows less than 40% toxicity induced by Cipa even at higher concentrations.

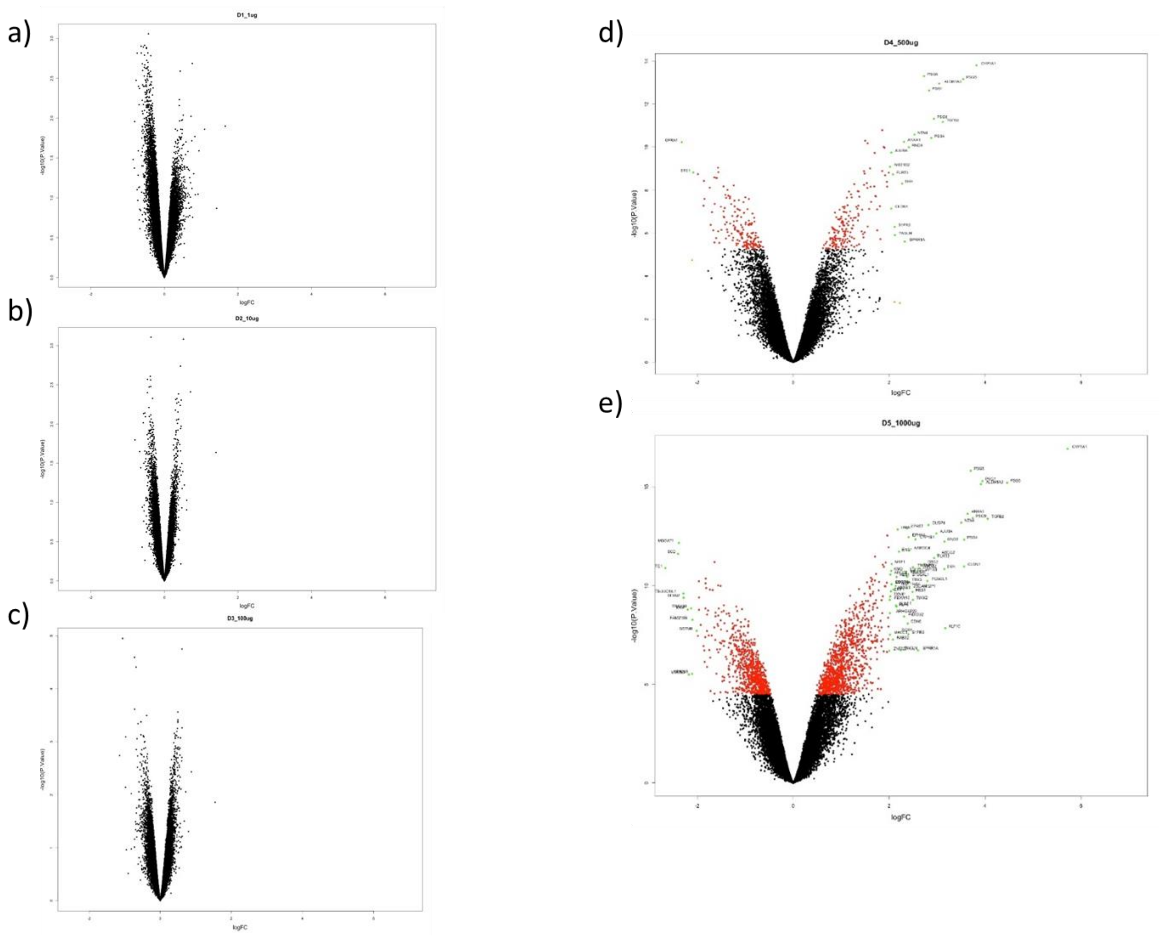

Fig S2. Volcano plots for probes with intensity value beyond the threshold for a-c) 1, 10 and 100μg, no probe qualifies the cut off, d) 500μg and e) 1000μg. p value<0.001, FC>|1|

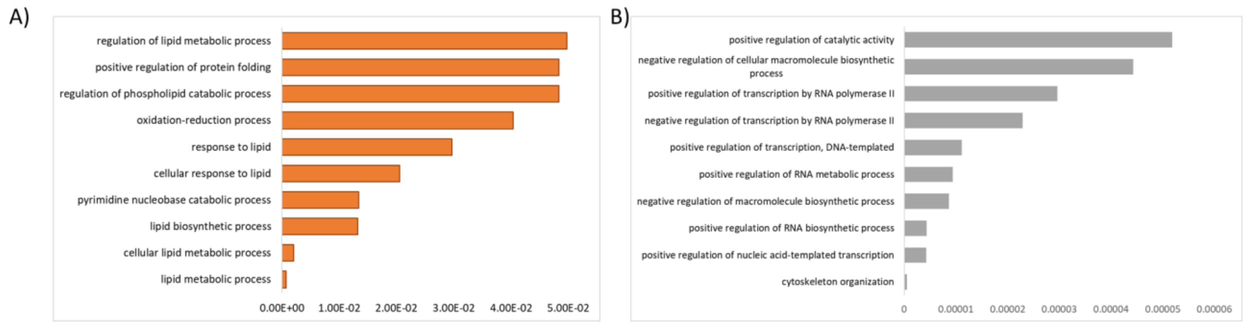

Fig S3. Gene ontology of the genes differentially expressed in response to Cipa. A) Biological pathways downregulated by Cipa. B) Biological pathways upregulated by Cipa. The p value was set to <0.01, FDR<0.05. This graph represents the biological processes from GO.

Regression with ERE sites	Coefficients	P-value
Intercept	0.725	4.756E-15
ERE sites 5KB Upstream	-0.004	0.018

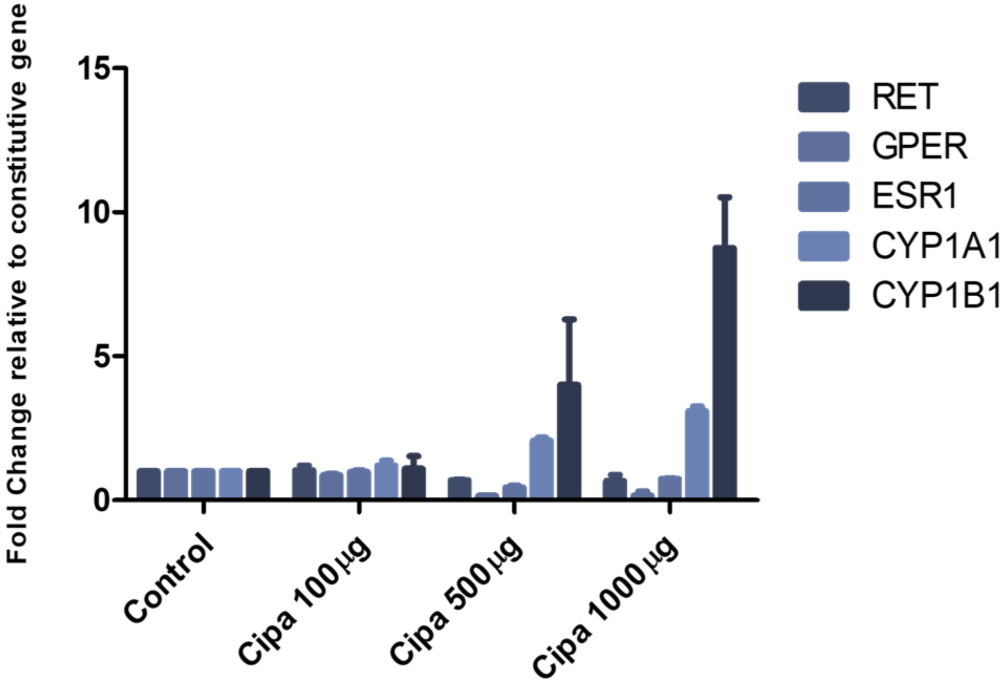

Fig S4. Regression values between fold change and the number of ERE sites. Response of well characterized estrogen responsive genes upon treatment of Cipa in MCF7 cells. Each of these genes have >25 ERE elements in their promoters.

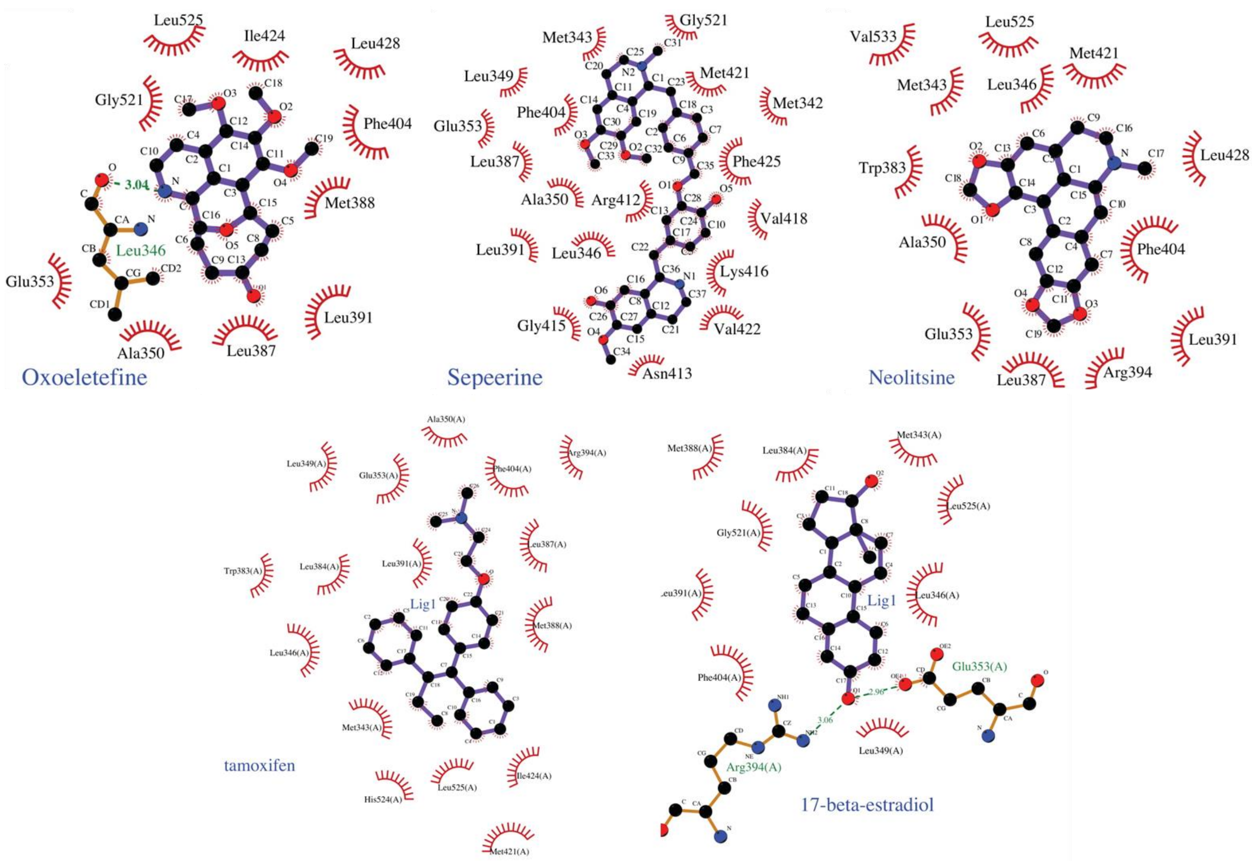

Fig S5. Docking details of the control molecules and the top three compounds constituent of Cipa around Erα.

a)

b)

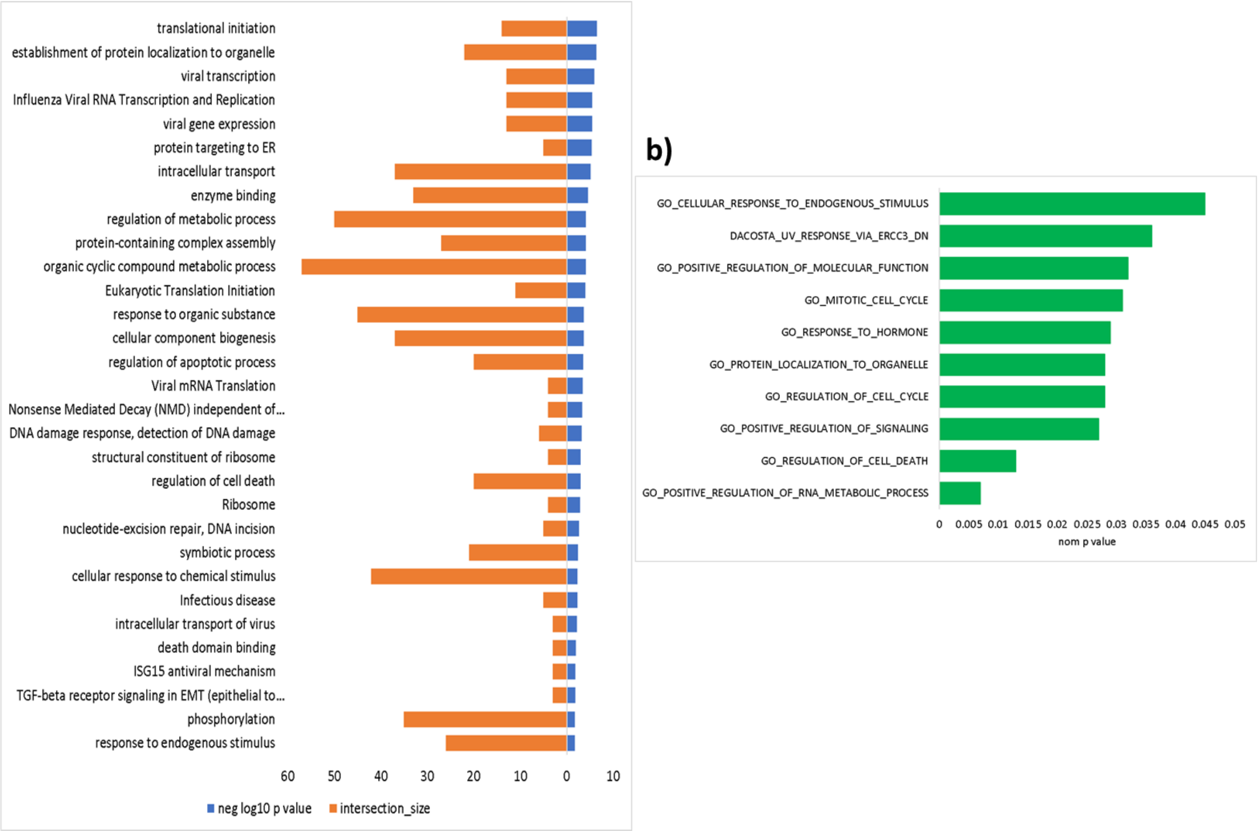

Fig S6. Terms enriched for genes with knockdown signature having >90 score connectivity with Cipa within CMAP (a) Biological pathways and processes and the number of genes intersecting with the total genes in the query (b) Gene sets enriched after GSEA.

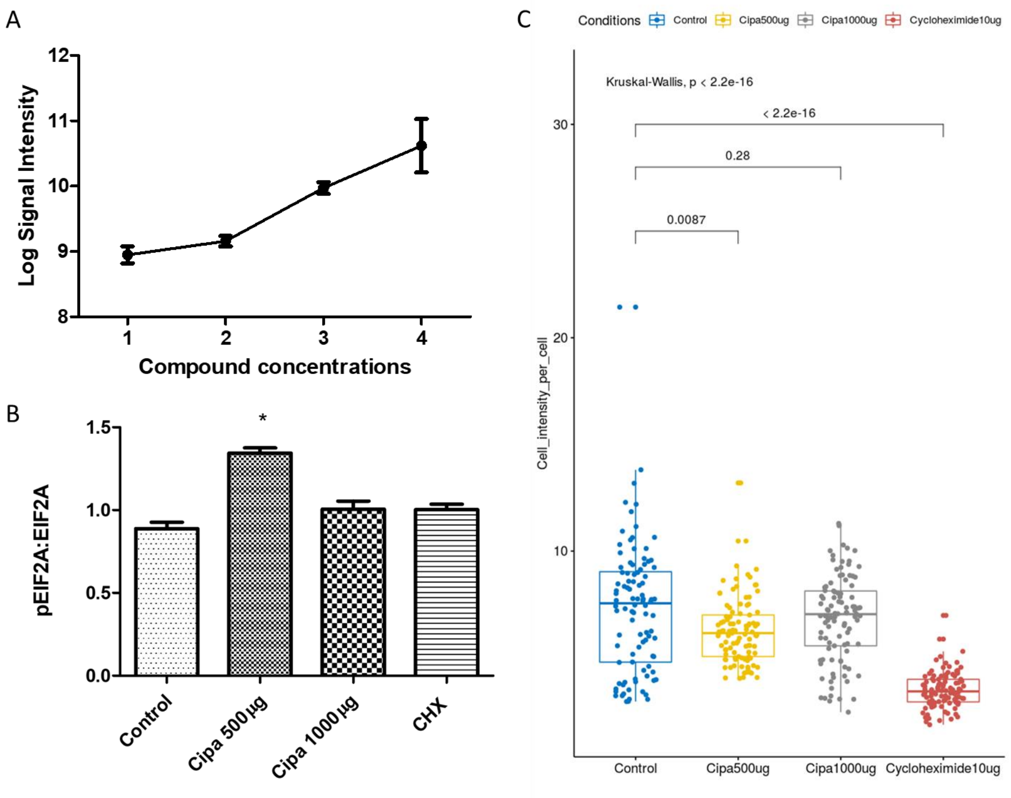

Fig S7. Cipa shows moderate protein translation inhibition: (A) EIF2AK3 microarray intensity values at the Cipa concentrations of 0, 100, 500 and 1000µg. (B) Ratio of phosphorylated EIF2A to non-phosphorylated EIF2A as determined by ICW. * p value=0.05 (C) Global protein translation inhibition determined using HPG assay. Fluorescent HPG replaces methionine during the formation of new proteins. The fluorescent values were measured at >100 different grid locations as intensity per grid and divided by the number of cells counted in the grid using high content imaging.

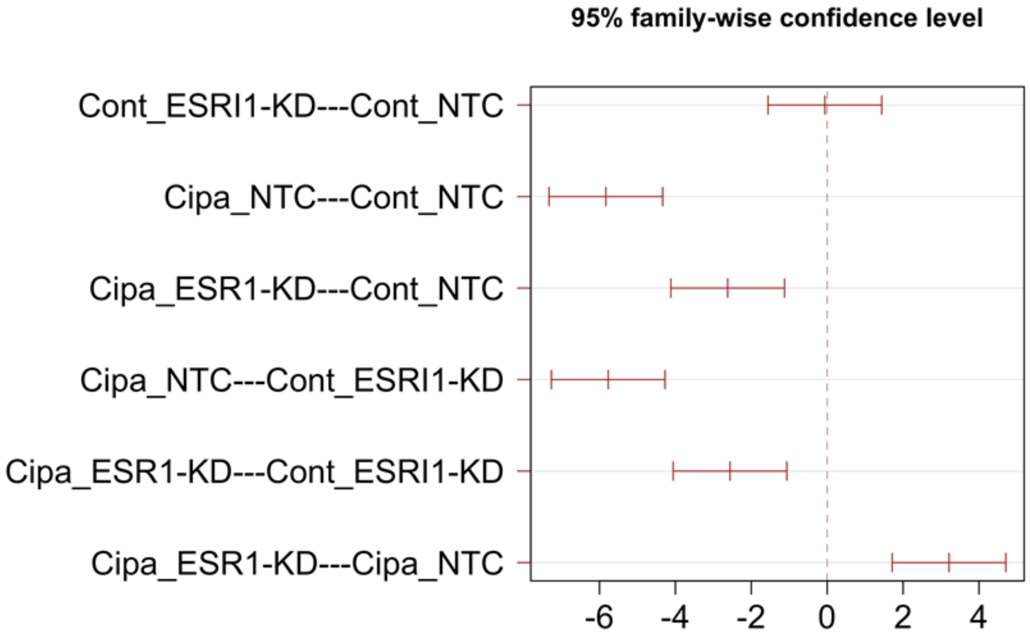

Fig S8. Difference in mean levels of viral titers. Tukey test compares the mean of every possible combination. Negative value indicates decrease in titers whereas positive value indicates that the mean (viral titer) is greater. Therefore, for conditions 1-5, there is an inhibition in the viral titers, however for the 6th condition, the inhibition is less compared to Cipa_NTC.

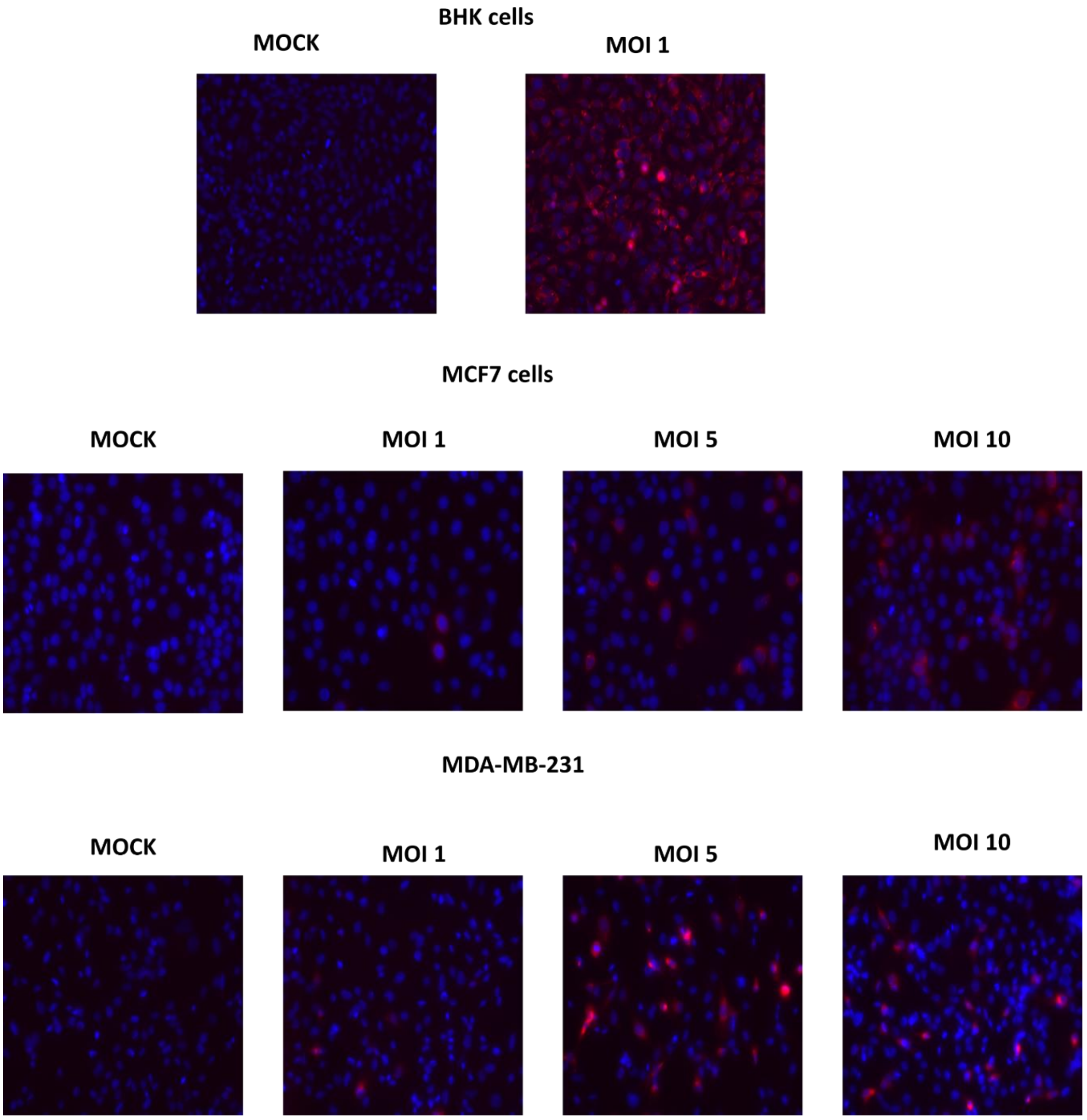

Fig S9. MOI of dengue virus in MCF7 and MDA-MB-231, the blue stains indicate the nuclei of the cells (DAPI) and the red stains indicate the viral proteins.

Table S1. Cipa constituent compounds, their minimum and maximum energies with ER-alpha, the binding cluster each fall into.

DRUG NAME	Minimum Binding Energy(kcal/mole)	Maximum Binding Energy (kcal/mole)	Cluster
**Sepeerine**	-10.2	-5.8	
**17-beta-estradiol (Control)**	-10.1		1
**Neolitsine**	-9.6	-5.5	1
**Oxoeletefine**	-9.6	-4.7	1
**Cycleanine**	-9.5	-7.5	1
**Liriodenine**	-9.5	-5.9	1
**Pareitropone**	-9.4	-5.2	1
**Tamoxifen (Control)**	-9.2		1
**Pronuciferine**	-9.1	-4.8	1
**Bulbocapnine**	-9	-5.4	1
**Cissaglaberrimine**	-9	-5.7	1
**Grandirubrine**	-8.8	-4.9	1
**Quercetin**	-8.8	-5.4	1
**Eletefine**	-8.7	-4.7	1
**Norruffscine**	-8.7	-5.2	1
**Pareirubrine_B**	-8.7	-5.1	1
**Oxobuxifoline**	-8.6	-5.7	1
**Trilobinine**	-8.6	-5.2	1
**Cissacapine**	-8.6	-6.9	
**Arachidic_acid**	-8.5	-5.7	1
**Cissamine**	-8.4	-5.1	1
**Magnoflorine**	-8.4	-5.2	1
**Norimeluteine**	-8.4	-4.8	1
**Methylwarifteine**	-8.4	-6.1	3
**Pelosine**	-8.4	-6	
**Corydine**	-8.3	-4.8	1
**Crotsparine**	-8.3	-4.9	1
**Glaziovine**	-8.3	-5	1
**Insulanoline**	-8.3	-6.2	2
**(S)-6-Methoxyjuziphine**	-8.3	-4.9	
**corytuberine**	-8.2	-5.2	1
**Cissampeloflavone**	-8.2	-5.9	2
**Nuciferine**	-8.1	-5	1
**Reticuline**	-8.1	-5	1

DRUG NAME	Minimum Binding Energy(kcal/mole)	Maximum Binding Energy (kcal/mole)	Cluster
**Roraimine**	-8.1	-6.1	3
**Milonine**	-8	-4.7	1
**N.N-Dimethyllindcarpine**	-8	-4.8	1
**Cissampentin**	-8	-6.2	3
**Hayatidine**	-7.9	-6.2	3
**Dimethylwarifteine**	-7.9	-5.7	4
**Hayatine**	-7.8	-6	3
**Laurifoline**	-7.7	-5.2	1
**Isochondrodendrine**	-7.7	-6.2	2
**Berberine**	-7.6	-5.2	2
**Cissampareine**	-7.6	-6.3	3
**Dehydrodicentrine**	-7.4	-5.4	1
**Dicentrine**	-7.4	-5.3	1
**Salutaridine**	-7.4	-4.6	1
**Hayatinin**	-7.4	-6	2
**Insularine**	-7.3	-5.8	2
**Reserpine**	-7.3	-5.8	2
**Warifteine**	-7.3	-5.4	4
**Isoimerubrine**	-7.2	-4.9	1
**Laudanosine**	-7.1	-4.7	1
**Deca-2E.4E-dienoic_acid_isobutylamide**	-6.7	-4	1
**Lauroscholtzine**	-6.7	-4.9	1
**Pareirubrine_A**	-6.7	-4.8	4
**Decene-2-oic_acid_isobutylamide**	-6.4	-3.7	1
**Decanoic_acid_isobutylamide**	-6.1	-3.4	1
**Thymol**	-6.1	-4.2	1
**Magniflorin**	-6.1	-4.9	2
**Galacturonic_acid**	-5.9	-4.3	
**d-Quercitol**	-5.7	-3.8	

Table S2: Antiviral activity of positively connected compounds

Compound	Virus	References	Year
Emetine	Coronavirus	PMID: 24841273	2014
	New Casle Disease, Bovine Herpervirus, Buffalopoxvirus	PMID: 28624461	2017
	Human Cytomegalovirus	PMID: 27336364	2016
	Rabies virus	PMID: 30028873	2018
	HIV-1	PMID: 26111177	2015
	Enterovirus	PMID: 31734270	2020
	HSV-2, HIV-1, Influenza	PMID: 31635418	2019
	Novel Coronavirus	PMID: 30918074	2020
	Zika and Ebola virus	PMID: 29872540	2018
Anisomycin	Coronavirus	PMID: 24841273	2014
	Poliovirus	PMID: 18243348	2008
	Dengue and Zika virus	PMID: 32081740	2020
Q-XII-47	Dengue virus	PMID: 28034743	2017
	Novel Coronavirus	*Preprint*	2020
Homoharringtonine	Coronavirus	PMID: 24841273	2014

	Vesicular stomatitis virus, Newcastle disease virus	PMID: 30388805	2018
	Coronavirus	PMID: 25451075	2015
	Hepatitis B virus, Bovine diarrhoea virus	PMID: 17458779	2007
	Foot and mouth disease virus	PMID: 31032977	2019
Cycloheximide	HIV-1, influenza viruses, coxsackie B virus, EV71	PMID: 21351439	2010
	Coronavirus	PMID: 21430047	2011
	Coronavirus	PMID: 24841273	2014
	Coronavirus	PMID: 21430047	2011
	Foot and mouth disease virus	PMID: 15649564	2005
	Human T-lymphotropic virus	PMID: 22214262	2012
	Adenovirus 1,2, 5	PMID: 11562975	2001
Cephaeline	Zika and Ebola virus	PMID: 29872540	2018

Table S3: Amounts (mg/g) of compounds quantified in different samples of *C. pareira*

**S. No.**	**Sample**	**Magnoflorine** (US-CP-7) **(1)**	**Pareirarine (2)**	**Salutaridine** (US-DR-CP-2) **(3)**	**Cissamine** (US-CP-3) **(4)**	**Hayatinine** (US-50) **(5)**	**Total alkaloids**
1.	USCPWP-PE-50	12.9	5.4	NQ	18.6	9.5	46.4
2.	USCPR-PE-R	3.5	1.6	NQ	6.0	1.8	12.9
3.	USCP-PE	NQ	NQ	NQ	3.3	NQ	3.3
4.	USCP-PE-EF	NQ	7.6	NQ	8.0	NQ	15.6
5.	USCP-PE-BF	NQ	10.3	NQ	1.7	NQ	12.0
